# Fluoroscopy-free Transcatheter Atrial Septal Defect Closure: A Simplified Approach

**DOI:** 10.1007/s11886-024-02177-5

**Published:** 2025-02-19

**Authors:** Naychi Lwin, Piia Suursalmi, Sophia Yong, Saleha Kabir, Matthew I Jones, Alexandra Savis, Shakeel A Qureshi, Eric Rosenthal

**Affiliations:** Department of Paediatric Cardiology, Evelina London Children’s Hospital, London, UK

**Keywords:** Atrial septal defect, Transcatheter closure, Fluoroscopy-free, Transoesophageal echocardiography, Congenital heart disease

## Abstract

**Purpose of Review:**

To provide an overview of fluoroscopy-free transcatheter atrial septal defect (ASD) closure and introduce a simplified approach that avoids pulmonary vein instrumentation.

**Recent Findings:**

Since the first reported fluoroscopy-free ASD closure 24 years ago, only a few small series have described this technique. We present a simplified and less cumbersome approach to encourage wider adoption of the fluoroscopy-free method to suitable ASD anatomy.

**Results:**

Fluoroscopy free ASD closure was performed in 9 patients using the conventional technique (Group 1) and 23 patients using our simplified approach of direct placement of the device into the defect (Group 2). Median age and weight were 28 years, 53 kg in Group 1 (range: 5–52 years, 22–88 kg) and 36 years, 66 kg in Group 2 (range: 4–76 years, 16–115 kg). Devices were successfully implanted in all patients, with a median device size of 21 mm (Group 1: 9–36 mm, Group 2: 10–33 mm). Procedural time was 47 min for Group 1 and 35 min for Group 2 (*p* = 0.09). Length of hospital stay was similar in both groups. There were no acute or long-term complications and no need for reintervention.

**Summary:**

Transcatheter ASD closure without the use of fluoroscopy using the simplified approach is safe and effective, offers a shorter procedure duration and minimises instrumentation within the left atrium and pulmonary veins. Patient selection is key and with greater experience, this procedure may be applicable to a wider selection of ASD anatomy.

**Supplementary Information:**

The online version contains supplementary material available at 10.1007/s11886-024-02177-5.

## Introduction

Atrial septal defects (ASD) are one of the most common congenital heart defects with an incidence reported at around 2.9 per 1000 live births [1]. The indications for ASD closure include symptoms or evidence of right ventricular volume overload in the absence of irreversible pulmonary hypertension [2, 3]. Transcatheter closure of ostium secundum atrial septal defects (ASD) using a combination of fluoroscopy and transoesophageal echocardiography has been the gold standard since the introduction of interventional ASD closure in the 1970s. This revolutionised the management of ASDs which had been predominantly a surgical procedure [4, 5]. When the defect anatomy is favourable, it is preferred to surgery due to the low morbidity, avoidance of a sternotomy, faster recovery time and reduced length of hospital stay [3, 6]. The procedural steps for transcatheter ASD closure begin with obtaining femoral venous access, followed by crossing the atrial septum and positioning a catheter in a left-sided pulmonary vein. A stiff, exchange length guidewire is then advanced into the pulmonary vein, which facilitates balloon interrogation of the defect (if necessary) and placement of a delivery sheath. An appropriately sized device is selected and deployed [7]. Systemic heparinisation and prophylactic antibiotics are typically administered at the beginning of the procedure, and the procedure is performed under transoesophageal or intracardiac echocardiographic guidance, with fluoroscopic assistance. Currently, there is an extremely low rate of adverse event in the hands of experienced operators however reported complications include transient exacerbation of migraine headaches in some patients, acute cardiac perforation, varying degrees of heart block, paroxysmal (usually transient) atrial fibrillation in older patients, device embolisation, cardiac erosion (hypothesised to be more common in oversized devices in patients with absent retroaortic rims), stroke and death [7–9]. In recent times, there have been increasing efforts to reduce the exposure of ionising radiation in the cardiac catheterisation laboratory to both patients and staff with greater appreciation of the harmful effects of radiation. This is particularly pertinent to infants and children due greater radiation sensitivity, large body surface area and expected life years [5, 10]. A recent study demonstrated over a 50% reduction in radiation use during ASD closure through quality improvement interventions (fluoroscopy guideline changes, enhanced monitoring, optimised TOE use, and improved communication with TOE staff) without impacting procedural success, duration, or acute complications [11]. The significant enhancements in 2D and 3D transthoracic and transoesophageal echocardiography for defect anatomy, defect size and rims, device and material visualisation have reduced the dependency on fluoroscopy [5, 12]. Diagnostic catheterisation is usually unnecessary and provides little additional information towards the decision for ASD closure provided non-invasive imaging is sufficient to estimate pulmonary artery pressures and shunt size [2, 13]. In our institution, diagnostic catheterisation is only performed to clarify discrepant or inconclusive non-invasive imaging data. Since the first reported transcatheter ASD closure without fluoroscopy twenty-four years ago [14], there have been several small series demonstrating the safety and feasibility of performing ASD closure using the traditional method of instrumenting the pulmonary vein with a wire, catheter and sheath, and deploying the ASD device exclusively under echocardiographic guidance. The intraprocedural outcomes from this approach including implant success rate, procedure time and complication rates have been comparable to fluoroscopy-guided closure [5, 6, 14–17] although many find this approach cumbersome.

In our institution, the first transcatheter ASD closure without fluoroscopy was performed in 2016, and over the last seven years, our procedural strategy has evolved. We have developed a more simplified approach, which involves deploying part of the ASD device within the superior vena cava (SVC), manoeuvring the device/sheath assembly across the ASD, and deployment of the device, as usual. This article describes our simplified approach and compares the two delivery methods of transcatheter ASD closure without the use of fluoroscopy.

## Methods

This was a retrospective, observational single centre study at the Guys and St Thomas’ NHS Foundation Trust Hospital. Between July 2016 and April 2024, there were a total of 380 standalone ASD closures, of which 29% were performed surgically. During this period, a total of 33 selected patients underwent fluoroscopy-free transcatheter ASD closure using either the conventional approach (Group 1) or the simplified superior vena caval approach (Group 2).

### Procedure characteristics

All procedures were performed in our congenital cardiac catheterisation laboratory under general anaesthesia. Intravenous heparinisation at 100 units/kg and prophylactic flucloxacillin or teicoplanin (in the case of penicillin allergy) were given as routine practice. Systematic transoesophageal echocardiography (TOE) assessment at 0^o^, 30^o^, 60^o^, 90^o^ and 120^o^ views were used to determine the size of the defect and assessment of rims for suitability for device closure. Patients who were considered to require balloon sizing of the ASD or had multiple defects were excluded from having a fluoroscopy-free procedure. In all the patients, only femoral venous access was obtained. A device size of 0–4 mm larger than the greatest diameter on TOE was chosen prior to percutaneous access.

*Group 1* patients had the procedure performed using the well-described technique similar to the normal fluoroscopy-guided ASD closure. A multipurpose catheter was manipulated across the ASD into the left atrium or pulmonary vein under TOE guidance. This was exchanged for an Amplatz 0.035” Extra-stiff guidewire (Cook Inc., Bloomington, IN, USA), which facilitated passage of the delivery sheath into the left atrium and pulmonary vein. The ASD device was deployed across the septum in the traditional way under TOE guidance.

**Fig. 1:**
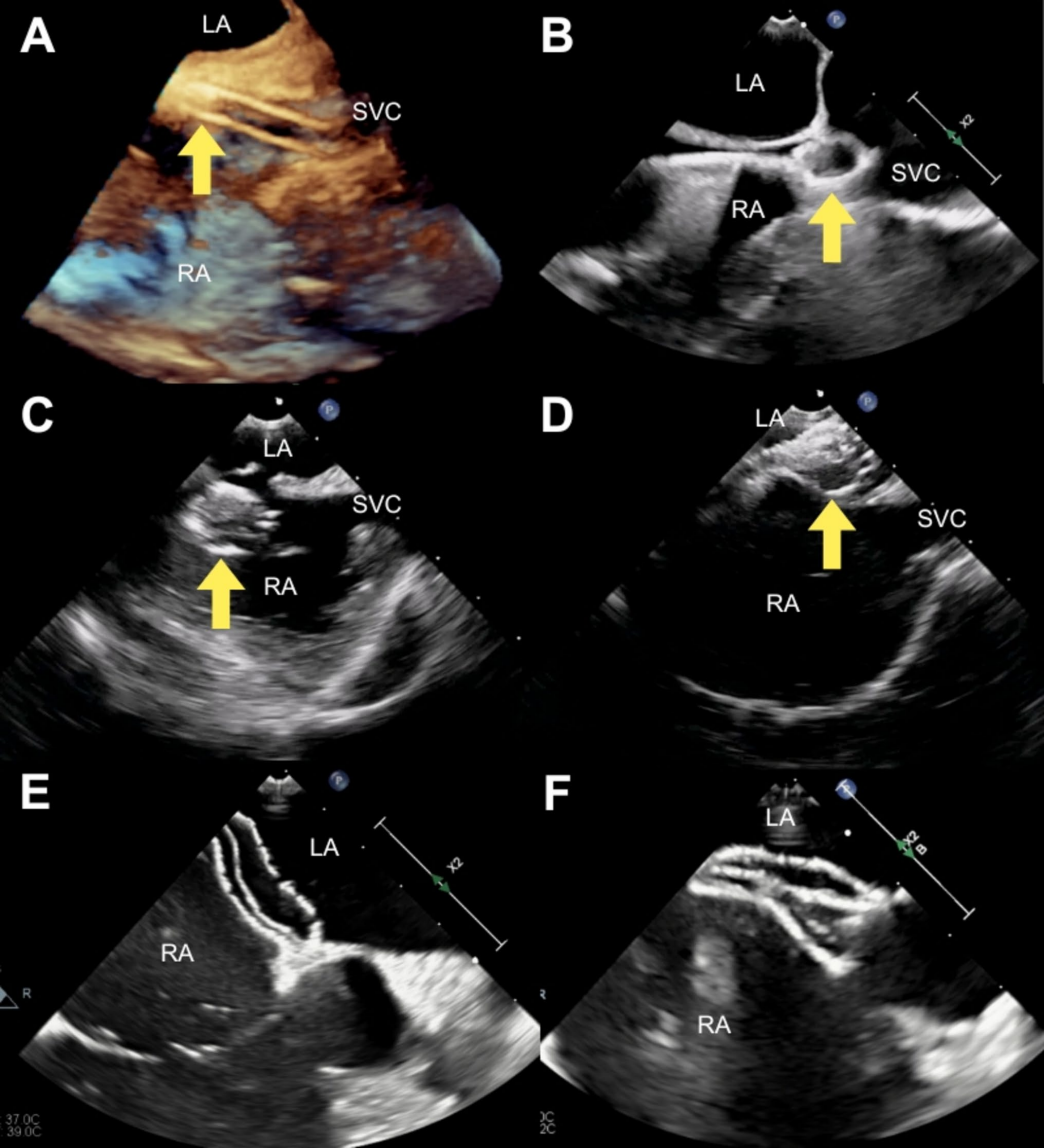
The modified procedure. (**A**) A 3D transoesophageal echocardiogram image shows the ASD occluder (arrow) advanced within the delivery sheath in the SVC. (**B**) A small portion of the left atrial disc of the occluder (arrow) is extruded from the end of the sheath in the SVC to form an ‘onion’. (**C**) The ‘onion’ and delivery sheath assembly (arrow) is rotated towards the ASD. (**D**) The ‘onion’ and delivery sheath assembly (arrow) is manoeuvred across the defect into the left atrium. (**E**) The left atrial disc is deployed and positioned. (**F**) The right atrial disc is deployed in the usual way. Key: SVC: superior vena cava; RA: right atrium, LA: left atrium

*Group 2* patients underwent a modified procedure (Fig. 1), whereby an Amplatz 0.035” Extra-stiff J-tip guidewire was advanced directly to the SVC followed by an appropriately sized sheath for the delivery of the device (Video 1). The wire and dilator were removed, and the selected septal occluder was advanced through the sheath, so that a small portion of the left atrial disc was extruded from the end of the sheath in the SVC to form an ‘onion’, easily visible on TOE (Video 2). The delivery sheath/device assembly was then withdrawn and manoeuvred across the ASD into the left atrium (Video 3) and the device was deployed and released in the usual way.

An Occlutech Figulla Flex II (Occlutech International, Helsingborg, Sweden) was used in 26 patients (9/9 in Group 1, and 17/24 in Group 2), Amplatzer Septal Occluder (Abbott Medical, St. Paul, MN, USA) in 5 patients (in Group 2), Cocoon Septal Occluder (Vascular Innovations Co., Nonthaburi, Thailand) in 1 patient (in Group 2) and CeraFlex ASD Closure System (Lifetech, Shenzhen, China) in 1 patient (in Group 2).

Final assessment was made with TOE including assessment of stability with a push-pull manoeuvre (the Minnesota wiggle) and the device was released. Haemostasis was achieved either by manual compression or Perclose ProStyle suture (Abbott Medical, St. Paul, MN, USA) depending on patient suitability and operator choice. A transthoracic echocardiogram was performed prior to discharge to assess immediate procedural success and exclude any complications. All patients received aspirin 5 mg / kg (maximum dose 75 mg daily) for 6 months and the initial follow-up was conducted after 2 months.

### Ethics and statistical analysis

This retrospective analysis was approved by the Guys & St Thomas NHS Trust Audit Committee. All patients, parents or legal guardians granted their informed consent for the procedure.

Statistical analysis was performed in Stata 18.0. Chi-squared tests was used for categorical variables and student t test or Mann Whitney-U was used for numerical variables. A *p*-value < 0.05 was considered statistically significant.

## Results

### Baseline clinical characteristics

Nine patients underwent fluoroscopy-free ASD closure using the conventional technique (Group 1) and 24 using our simplified approach (Group 2). There was no statistically significant difference in the demographic characteristics of the two groups (Table 1). The median age of the patients was 28 years (range 5–52 years) and 36 years (range 4–76 years) respectively. The median weight was 53 kg (range 22–88 kg) for the group 1 patients and 66 kg (range 16–115 kg) for group 2. Children were defined as less than 18 years of age. There were proportionately more children in group 1 (44% compared with 33% in Group 2), however this was not significant (*p* = 0.41). There were 44% males in Group 1 compared with 33% in Group 2. Comorbidities in six patients included biliary obstruction (resolved after biliary intervention), insulin intolerance, a regurgitant bicuspid aortic valve and developmental delay, obesity in two and migraine.

**Table 1:** Patient characteristics

	Group 1 (Traditional approach) (n = 9)	Group 2 (SVC approach) (n = 24)	*p *value
Age (years)			
Median [IQR]	28 [9 – 39]	36 [13 – 57]	
Range	5 – 52	4 – 76	0.21
Child	4 (44%)	7 (29%)	0.41
Weight (kg)			
Median [IQR]	53 [29 – 73]	66 [36 – 88]	
Range	22 – 88	16 – 115	0.38
Male	4 (44%)	8 (33%)	0.55

Key:SVC: superior vena cava, IQR: interquartile range.

### ASD characteristics

A single ostium secundum atrial septal defect was present in all the patients. The median defect size was 17 mm (range 9–36 mm) in Group 1 compared with 18 mm (range 10–28 mm) in Group 2. There was at least one deficient rim in 22/33 patients (67% in Group 1 and 67% in Group 2). The aortic rim was deficient in all those patients who had at least one deficient rim. One child (5 years, 25 kg) in Group 1 with a 13 mm defect and two deficient rims (aortic and superior vena cava) had successful transcatheter occlusion with a 15 mm device via the pulmonary venous approach. The largest defect size was 36 mm (in a 52 year old, 76 kg adult) and was successfully closed with a Occlutech Figulla Flex II 36 mm deployed via the pulmonary venous approach. The characteristics of the ASDs or the chosen device sizes were not significantly different in the two groups (Table 2).

**Table 2:** ASD characteristics and procedural details

	Group 1 (Traditional approach) (n = 9)	Group 2 (SVC approach) (n = 24)	*p *value
Defect size (mm)			
Median [IQR]	17 [15 – 20]	18 [16 – 21]	
Range	9 – 36	10 – 28	0.61
Device size (mm)			
Median [IQR]	21 [18 – 24]	21 [20 – 25]	
Range	9 – 36	10 – 33	0.61
Deficient Rim	6 (67%)	16 (67%)	0.25
Sheath size (Fr)			
Median [IQR]	9 [9 – 11]	11 [10 – 12]	
Range	7 – 12	7 – 14	0.15
Procedure time (min)			
Median [IQR]	47 [32 – 50]	35 [29 – 42]	
Range	21 – 65	20 – 51	0.09
Length of Stay (range, days)	1 (1 – 3)	1 (0 – 2)	0.30
Duration of Follow-Up (months)			
Median [IQR]	54 [23 – 66]	16 [7 – 47]	
Range	2 – 83	0 – 199	0.17
Major Adverse Events (including device embolisation, cardiac perforation, rhythm disturbance, stroke, death)	0	0	
Minor Adverse Events (transient migraine)	3	1	
Residual Shunt	0	0	

Key**:** ASD: atrial septal defect, SVC: superior vena cava, IQR: interquartile range, Fr: French.

### Procedural Outcomes

The device was successfully implanted in all the patients in whom closure was attempted. The median device size used was 21 mm in both groups (range 9–36 mm in Group 1, 10–33 mm in Group 2) (*p* = 0.61). The median sheath size used in Group 1 was 9 French (range 7–12 French) compared with 11 French (range 7–14 French) in Group 2 (*p* = 0.15). There was a shorter procedure time in Group 2 at 35 min (range 20–51 min) compared with Group 1 at 47 min (range 32–50 min) although this was not significant (*p* = 0.09). The median length of stay was 1 day (1–3 days in Group 1 compared with 0–2 days in Group 2). Within our institution, there were a total of 335 transcatheter ASD closures (302 performed with fluoroscopy and 33 without fluoroscopy assistance). The median procedure time in the group using fluoroscopy was 50 min compared with 37 min in the group without fluoroscopy (*p* < 0.001). This difference could however be confounded by difference in complexity of the procedure in each group.

### Patient Outcomes

 There were no intraprocedural, or early or late complications, and no need for reintervention. Median follow-up in Group 1 was 54 months (range 2–83 months) compared with 16 months (range 0–199 months) (*p* = 0.17) with shorter follow-up duration in Group 2 attributable to the change in procedural practice. Follow-up information as not available for 1 patient due to the short duration since the procedure. All the other patients had no residual shunt at the latest follow-up. One patient in whom there was a trivial shunt on the discharge echocardiogram, had no shunt at follow-up. Four patients experienced migraine (one pre-existing), three immediately post-procedure and one at three weeks post-procedure with spontaneous resolution.

## Discussion

In recent years, it has become imperative to minimise radiation exposure to both patient and staff working with ionising radiation. Ewert et al. reported the first series of four patients who underwent transcatheter ASD closure without the use of fluoroscopy [14]. Since this time, there have been seven small to medium sized series of transcatheter ASD closure in both adults and children without fluoroscopy confirming the feasibility of this approach [5, 6, 12, 14–19]. The described approach mimics the fluoroscopic procedure by instrumenting the pulmonary vein with a wire and delivery sheath, and deployment of the device in the usual way. Two groups required modification of their delivery sheath to permit their fluoroscopy free approach [15, 18]. Depending on institutional preference, balloon sizing without fluoroscopy has also been described from several centres [5, 20]. A recent study comparing long-term outcomes and costs found that TOE-guided percutaneous ASD closure in children resulted in shorter procedural times, fewer complications, and lower costs than the traditional fluoroscopic approach [19]. Fluoroscopy-free device closure of ASDs has been performed predominantly with transoesophageal echocardiogram however, ASD closure with transthoracic echocardiogram alone has been described in three series from Asia of 60, 20 and 14 patients with two of these three groups having the procedure performed under intravenous sedation only [12, 16, 18]. Comparisons between TTE and TOE will be needed with regards to anatomy visualisation, defect sizing and choice of devices but this certainly offers advantages to the fluoroscopy-free approach.

Despite the evidence supporting its technical feasibility and benefits, the majority of transcatheter ASD closures are still performed using fluoroscopy. In our institution, we strongly advocate fluoroscopy-free ASD closure in carefully selected patients with suitable ASD anatomy. Within our cohort, we selected ostium secundum atrial atrial defects and excluded defects that were multiple or fenestrated, that required balloon sizing (if there was a question about defect size or rim stability on echocardiogram), and procedures that required additional diagnostic assessment or interventional procedures. The shorter procedure time observed in patients undergoing fluoroscopy-free ASD closure, compared to traditional fluoroscopic closure, may also be attributed to the selection of less complex patients. Good communication and working relationship with experienced and trained echocardiographers performing the transoesophageal echocardiogram is essential for facilitating the procedure. We continue to perform ASD closure in a catheterisation laboratory with fluoroscopy available, in case of unexpected complications that necessitate fluoroscopy. However, with greater experience, this may become superfluous. During our 7-year experience of performing fluoroscopy-free transcatheter ASD closure, we have adapted our technique from a traditional pulmonary vein approach to a more simplified one avoiding the need for pulmonary vein access. It minimises the time required for equipment within the left atrium, reducing the risk of trauma and thrombus formation in and around the sheath. We noticed a decrease in the procedure time with this approach and anticipate this will become more streamlined with greater experience.

## Conclusion

Transcatheter ASD closure without the use of fluoroscopy is safe and effective. This small series describes the successful outcomes of a novel simplified approach without complications and a shorter procedure time in selected patients.

We recommend operators to select patients requiring ASD closure in whom these approaches may be possible to minimise radiation. This simplified and less cumbersome approach may encourage operators to employ the fluoroscopy-free approach to suitable ASD anatomy. With greater experience, this procedure may be applicable to a wider range of patients and ASD anatomy.

## Key References

- Harrison DJ, Shirley L, Michaud J, Rivera J, Quinn B, Bergersen L, et al. The burden of radiation exposure during transcatheter closure of atrial septal defect. The American Journal of Cardiology. 2021;149:126 − 31.
  - *Efforts to reduce ionizing radiation in the cardiac catheterization laboratory have increased. Findings from this study suggests the potential for over a 50% reduction in patient radiation exposure with the implementation of quality improvement interventions.*
- Ke Q, Weng G, Xie Q, Bao J, Zheng F, Huang J, et al. Comparison of Long-Term Clinical Outcomes and Costs Between Transesophageal Echocardiography-Guided and X-ray-Guided Percutaneous Atrial Septal Defect Closure in Children. Pediatric Cardiology. 2024:1–8.
  - *Findings from this study suggests that in addition to reducing radiation exposure to both patients and staff*,* the fluoroscopy-free approach resulted in shorter procedural times*,* fewer acute complications and lower procedural costs.*

## Supplementary Information

Below is the link to the electronic supplementary material.

ESM 1 A 3D transoesophageal echocardiogram image shows the atrial septal defect occluder advanced within the delivery sheath in the superior vena cava.(MP4 860 KB)

ESM 2 A small portion of the left atrial disc of the occluder is extruded from the end of the sheath in the superior vena cava to form an ‘onion’.(MP4 1.02 MB)

ESM 3 The ‘onion’ and delivery sheath assembly is rotated towards the atrial septal defect. The ‘onion’ and delivery sheath assembly is manoeuvred across the defect into the left atrium.(MP4 4.95 MB)

## Data Availability

No datasets were generated or analysed during the current study.

## Abbreviations

ASD: atrial septal defect
SVC: superior vena cava
TOE: transoesophageal echocardiogram
